# Editorial: Neuroimaging of brain structure-function coupling mechanism in neuropsychiatric disorders

**DOI:** 10.3389/fnins.2023.1270645

**Published:** 2023-08-14

**Authors:** Mengmeng Feng, Hongwei Wen, Jing Li, Han Lv, Junghun Cho, Lingfei Guo

**Affiliations:** ^1^Department of Radiology, Shandong Provincial Hospital, Shandong University, Jinan, Shandong, China; ^2^Key Laboratory of Cognition and Personality (Ministry of Education), Faculty of Psychology, Southwest University, Chongqing, China; ^3^Department of Radiology, Beijing Friendship Hospital, Capital Medical University, Beijing, China; ^4^Department of Biomedical Engineering, University at Buffalo, The State University of New York, New York, NY, United States; ^5^Key Laboratory of Endocrine Glucose and Lipids Metabolism and Brain Aging, Ministry of Education, Jinan, Shandong, China; ^6^Department of Radiology, Shandong Provincial Hospital Affiliated to Shandong First Medical University, Jinan, Shandong, China

**Keywords:** neuropsychiatric disorders, multimodal MRI, structure-function coupling, neurovascular coupling, function impairment

Neuropsychiatric disorders are diseases with alterations in the nervous system or mental activity disorders, with cognitive and motor disorders and behavioral changes as the core, mainly including Parkinson's disease (PD), Alzheimer's disease, Huntington's disease, narcolepsy, schizophrenia, Tourette syndrome and autism spectrum disorders (ASD) (Dugger and Dickson, 2017). With the development of multimodal magnetic resonance imaging (MRI) neuroimaging methods, the underlying neurobiological mechanisms of neuropsychiatric disorders have been increasingly investigated, and the brain structure-function coupling approach has received more focused attention in recent years because it can enhance sensitivity to detect brain pathophysiological abnormalities compared to single functional or structural indicators (Sarwar et al., 2021). In addition, neurovascular coupling (NVC), as an important mechanism for neurogenic regulation of cerebral blood flow (CBF), also plays an important role in neuropsychiatric diseases, and alterations in NVC can also cause structure-function coupling changes, which can lead to neurocognitive aging and behavioral disorders (Kisler et al., 2017). The purpose of this Research Topic is to investigate the structural and functional changes, especially coupling changes, in patients with neuropsychiatric disorders, and their correlation mechanisms with cognitive/motor/other functional parameters, in conjunction with multimodal MRI techniques and thus provide the theoretical basis for clinical diagnosis and treatment. The authors focused not only on the common neuropsychiatric disorders mentioned above, but also on other neuropsychiatric disorders that affect the quality of life and survival of pregnant women, children, and adults, and explored the mechanisms involved in the disorders from multiple perspectives, including structure, function, and metabolic, using multimodal MRI.

Many parameters can describe structure-function coupling in neuroimaging. Wang N. et al. systematically summarized the current multimodal MRI-based analysis techniques and coupling parameters from the perspectives of voxel-wise structure-function coupling, structural connectivity-functional connectivity coupling, and NVC in patients with cerebral small vessel disease (CSVD), providing an imaging perspective for studying the pathogenesis and early diagnosis of the disease. Some studies in this Research Topic obtain parameters such as amplitude of low-frequency fluctuation (ALFF), fractional amplitude of low-frequency fluctuation (fALFF), regional homogeneity (ReHo), degree centrality (DC), and functional connectivity strength (FCS) based on resting-state functional MRI (rs-fMRI), and obtain CBF maps based on 3D pseudo-continuous arterial spin labeling (3D pCASL). And they characterize NVC with the correlation between rs-fMRI parameters and CBF maps. Li X. et al. focused on medication-overuse headache (MOH) and revealed a significantly decreased NVC in the left orbit part of the superior frontal gyrus, the bilateral gyrus rectus, and the olfactory cortex, and a negative correlation between neuronal activity and CBF at the voxel level in MOH, implying a disturbance of normal physiological conditions. Yang et al. studied whole gray matter CBF-FCS coupling in Meige syndrome (MS) patients. They found the CBF-FCS coupling was increased, and the CBF values of the middle frontal gyrus and the bilateral precentral gyrus were significantly increased in MS patients, which indicates a compensated blood perfusion in motor-related brain regions and reorganized the balance between neuronal activity and brain blood supply. Ruan et al. found that NVC was significantly reduced at both whole-brain and brain region levels in small vessel disease cognitive impairment (SVCI) and post-stroke cognitive impairment (PSCI) patients. They also used mediation analysis to reveal the mediating role of NVC between white matter (WM) lesion burden and cognitive impairment. The potential of NVC in accurately measuring cognitive impairment cannot be underestimated, and it may guide targeted therapies aimed at improving cognitive function in this patient population in the future. Zhang, Liang et al. used ALFF and voxel-based morphometry (VBM) to define NVC and found that structure-function decoupling in the bilateral caudate nucleus has advantages in identifying patients with severe CSVD burden, which is helpful for early prediction and diagnosis in the development of CSVD. Understanding the regulatory mechanism of NVC in diseases is of great significance in preventing further disease development and guiding treatment.

The characteristically unilateral pattern of symptoms in PD at onset and early stages is considered an important diagnostic feature. To compare the differences in brain structure and function in PD patients with different onset sides, Zhang, Li et al. obtained the topology of functional and structural connectome based on rs-fMRI and diffusion weighted imaging (DTI) by graph theory. Ultimately, no difference was evident from the rs-fMRI- or DTI-derived network alone among patients with different sides of onset and HCs, but there were significant differences in network metrics of resting-state-informed structural connectome. Topological properties of motor-related brain networks could distinguish LPD and RPD, which provides neurobiological insights into the lateralization of PD onset. Lin et al. used the microstructural and functional neuroimaging from the Adolescents Behavior Cognitive Development (ABCD) database to comprehensively assess the underlying neural mechanisms of Attention-Deficit Hyperactivity Disorder (ADHD) in children. The results indicate a pervasive reduced microstructural integrity in WM in ADHD children, which may lead to impaired connectivity of attention and default mode functional networks. In addition, the authors used a machine learning approach to combine multiple structural and functional imaging metrics to validate different combinations of classification models to aid in the diagnosis of ADHD in clinical children. Bian et al. used machine learning to combine functional and structural connectivity parameters to predict motor impairment after stroke, which provides a theoretical basis for personalized treatment and effective rehabilitation. In addition, they also found that models utilizing functional connectivity tended to have better performance than those utilizing structural connectivity, in which the dorsal and ventral attention networks are important features. In general, these studies fit into our Research Topic theme of interpreting neural mechanisms from a structure-function perspective and have important implications for both mechanistic studies and clinical diagnosis, treatment, and prognostic assessment.

Although structure-function coupling is a more comprehensive approach to studying the mechanisms of neuropsychiatric disorders, independent analysis of structural or functional MRI methods can also facilitate in-depth exploration of disease mechanisms. Wang H. et al. studied the WM structural networks of High myopia (HM) patients based on diffusion kurtosis imaging (DKI) and topography. They found the local efficiency and clustering coefficient decreased significantly, the hub distributions were partially reorganized, and the node betweenness centrality also changed significantly in HM patients. The changes in the networks provided a new perspective for people to explore the mechanism. Guo et al. introduced Mendelian randomization study into the correlation analysis of brain structure and migraine to further explore whether there is a causal relationship. The results showed that the decreased cerebral cortical surface area and hippocampal volume are associated with higher migraine risk. It will be more helpful to elucidate the underlying mechanisms of migraine and will also provide new ideas for the subsequent treatment of migraine. A review of cobalamin C (cblC) defects in infants found ventricular dilation, cerebral atrophy, and corpus callosum thinning are the main MRI abnormalities of cblC defects, and these manifestations are significantly correlated with delayed development in children. MRI findings can be considered an important tool for determining the severity of cblC defects. Gao et al. proposed that gray matter volume (GMV) could be used as a potential biological indicator of type 2 diabetes mellitus (T2DM), and revealed the correlation between the GMV and serum P-tau-181 in T2DM patients. In future research, the role of P-tau-181 in diabetes encephalopathy cannot be ignored. At the same time, early detection and early intervention should be carried out for T2DM patients to prevent the disease from further worsening and causing more serious complications.

Li, Du et al. used rs-fMRI to assess altered spontaneous brain activity of overseas college students during the coronavirus 2019 pandemic. The MRI data showed altered ALFF and ReHo values in the superior medial frontal gyrus, precentral gyrus, and paracentral lobule in the overseas students and the spontaneous brain activity correlated with anxiety and depression. Focusing on patients with major depressive disorder (MDD), Liu et al. showed that short-term repeated treatment with ketamine altered resting-state functional connectivity (RSFC) of amygdala-related networks, suggesting that the baseline RSFC between bilateral amygdala and right putamen may be a predictor of the response of ketamine's antidepressant and antisuicidal, facilitating the clinical assessment of MDD. In addition, the ASL technique allows quantitative measurement of brain perfusion and is currently the most popular imaging technique for responding to tissue metabolism. Ye et al. used PCASL to compare regional CBF in ASD individuals with their age-matched typically developing (TD) children using pCASL perfusion imaging and to explore the relationship between CBF and clinical characteristics/developmental profile of ASD. The results indicated that CBF values are a potential MRI-based biomarker of disease severity in ASD patients. The above-mentioned articles explore the structural, functional, or perfusion alterations in specific brain regions of patients with neuropsychiatric disorders through different imaging methods and how these alterations lead to clinical symptoms, as well as providing a basis for future precise treatment of patients.

Several authors in this Research Topic focused on preeclampsia (PE), a condition specific to pregnant women. Zhang N. et al. reviewed PE-related reversible posterior leukoencephalopathy syndrome (RPLS), which is a serious threat to the health of pregnant women. The advantages and characteristic imaging manifestations of different current imaging methods, especially multimodal MRI, for the detection of PE-RPLS were summarized. Because the syndrome can lead to brain injury and has high morbidity and mortality rates, early detection, early diagnosis and prognostic assessment of RPLS can be achieved through ever-advancing imaging techniques. In addition, Wang Y. et al. investigated the changes in cognitive function and serum indicators (P-tau181 protein and T-tau protein) of PE patients during pregnancy. The results showed that PE patients had a certain degree of cognitive impairment, as the cognitive test scores of PE patients were lower than those of the pregnant healthy controls and non-pregnant healthy controls. At the same time, the concentration of serum P-tau181 had a great correlation with the cognitive test scores. Thus, serum P-tau181 protein concentration has potential value in the diagnosis of cognitive impairment in PE patients, which can serve as a simple, accessible, and scalable marker for the screening and diagnosis of cognitive functional impairment in PE. Sui et al. found the local GMV in the right middle temporal gyrus will be significantly reduced, which affected the language motor function and cognitive flexibility in PE superimposed on chronic hypertension patients. These studies highlight the importance of brain damage and cognitive decline in pregnant women with PE, and multimodal MRI methods and serology can be used for early diagnosis and treatment of the disease.

The Research Topic also includes two review reports. Li, Bao et al. reviewed the neuroimaging mechanisms of heart failure (HF)-induced cognitive impairment from MRI perspective and summarized the key roles of decreased cerebral perfusion pressure, an inflammatory response, oxidative stress, and BBB breakdown in the development of cognitive impairment and changes in the structural, functional, and metabolic status of the brain in HF patients. Emerging MRI techniques allow non-invasive detection of functional and structural changes in the brain, which can help further investigate early pathophysiological changes in HF patients with CI. Another review focused on methylmalonic acidemia (MMA), which affects the survival rate and quality of life of newborns or infants, and summarized the neuroimaging features of MMA. The accumulation of methylmalonic acid and other metabolites in the body of patients causes brain tissue damage, which can manifest as various degrees of intellectual disability and severe neurological dysfunction. MRI functional brain imaging can assess the development of the brain and the degree of brain damage, and derive a qualitative diagnosis, which provides an objective basis for early clinical diagnosis and also helps to determine the prognosis and improve the quality of life of patients.

In summary, multimodal MRI plays an increasingly important role in the study of the mechanisms of neuropsychiatric diseases. Obtaining structure-function coupling parameters through multimodal MRI has become a new tool for exploring disease mechanisms, early diagnosis, guiding treatment, and evaluating prognosis. In addition, a single structural or functional study cannot be ignored, and the development of any single technology will drive the development of coupling. The papers in this Research Topic also demonstrated the potential for structure-function coupling in the future.

## Author contributions

MF: Writing—original draft, Writing—review and editing. HW: Writing—original draft, Writing—review and editing. JL: Writing—original draft, Writing—review and editing. HL: Writing—original draft, Writing—review and editing. JC: Writing—original draft, Writing—review and editing. LG: Writing—original draft, Writing—review and editing.
